# Revolutionizing mRNA Vaccines Through Innovative Formulation and Delivery Strategies

**DOI:** 10.3390/biom15030359

**Published:** 2025-03-01

**Authors:** Munazza Fatima, Timothy An, Kee-Jong Hong

**Affiliations:** 1 Department of Microbiology, Gachon University College of Medicine, Incheon 21936, Republic of Korea; munazzafatima@gachon.ac.kr; 2 Lee Gil Ya Cancer and Diabetes Institute, Gachon University, Incheon 21999, Republic of Korea; 3 Department of Health Sciences and Technology, GAIHST, Gachon University, Incheon 21999, Republic of Korea; 4 Korea mRNA Vaccine Initiative, Gachon University, Seongnam 13120, Republic of Korea

**Keywords:** lipid nanoparticles, mRNA delivery, polymeric nanoparticles, liposomes, extracellular vesicles, dendrimer, hybrid nanoparticles, DNA nanostructures

## Abstract

Modernization of existing methods for the delivery of mRNA is vital in advanced therapeutics. Traditionally, mRNA has faced obstacles of poor stability due to enzymatic degradation. This work examines cutting-edge formulation and emerging techniques for safer delivery of mRNA vaccines. Inspired by the success of lipid nanoparticles (LNP) in delivering mRNA vaccines for COVID-19, a variety of other formulations have been developed to deliver mRNA vaccines for diverse infections. The meritorious features of nanoparticle-based mRNA delivery strategies, including LNP, polymeric, dendrimers, polysaccharide-based, peptide-derived, carbon and metal-based, DNA nanostructures, hybrid, and extracellular vesicles, have been examined. The impact of these delivery platforms on mRNA vaccine delivery efficacy, protection from enzymatic degradation, cellular uptake, controlled release, and immunogenicity has been discussed in detail. Even with significant developments, there are certain limitations to overcome, including toxicity concerns, limited information about immune pathways, the need to maintain a cold chain, and the necessity of optimizing administration methods. Continuous innovation is essential for improving delivery systems for mRNA vaccines. Future research directions have been proposed to address the existing challenges in mRNA delivery and to expand their potential prophylactic and therapeutic application.

## 1. Introduction

Messenger RNA (mRNA) was discovered in 1961 [1]. In vitro transcription (IVT) of mRNA appeared as a significant milestone to extend the application of mRNA in therapeutics [2]. Advancements in this field were hampered by various challenges, including immunogenicity, instability, and expensive mRNA processing. The development of innovative strategies such as chemical modifications [3], sequence optimization [4,5], and purification of mRNA [6] has significantly impacted vaccine delivery and efficacy. Despite notable advancements in mRNA engineering, obstacles like low cellular uptake, instability, and ineffective delivery prevent it from being used in a wider range of clinical settings. The negative charge of mRNA originates from phosphate moieties, which make it difficult to cross through the anionic cell membrane. Furthermore, due to its intrinsically unstable nature, mRNA is vulnerable to enzymatic breakdown in biological settings, which reduces its therapeutic effect [7]. Precipitation or aggregation of mRNA may reduce its translation, resulting in lower protein production. Moreover, degraded fragments of mRNA may lead to undesired immune response, adversely affecting the efficacy of mRNA vaccines [8]. Because of these limitations, higher dosages of mRNA are needed to achieve the required immune response [8]. The mRNA vaccine induces immune responses through pattern recognition receptors (PRRs), which include Toll-like receptors (TLRs), RIG-I-like receptors (RLRs), and NOD-like receptors (NLRs). These receptors play an important role in activating innate immune responses [9]. The TLRs found in endosomes or cell membranes can detect mRNA; their activation produces type I interferons and pro-inflammatory cytokines [10]. RIG-I detects mRNA in the cytoplasm, and their activation also leads to the production of interferons type I [11]. It is important to recognize that type I interferons play a key role in the immune response induced by mRNA vaccines. Additionally, a cytoplasmic NOD-like receptor activates the RIPK2–NF-κB pathway in response to cellular stress, leading to the secretion of IL-1β and amplifying the inflammatory response [12]. These innate responses promote immune cell recruitment, antigen presentation, and activation of CD8+ T cells, CD4+ T cells, and B cells, which lead to antibody production and long-term immune memory, thereby establishing an adaptive immune response [13,14]. The mRNA needs to be tailored to activate innate immunity sufficiently to trigger adaptive immunity while avoiding over-activation, which may hinder effective translation of mRNA [14]. Hence, comprehension of immune activation of mRNA vaccines is important for designing next-generation mRNA vaccines. Regulating the innate and adaptive immune response is the existing key challenge in mRNA vaccines. Several strategies, such as nucleoside modification, removal of dsRNA contamination, and introducing adjuvants in LNP formulations, are being investigated to address these challenges [15].

Immense efforts are devoted to improving the immunogenicity, stability, and delivery of mRNA vaccines, which could extend their potential for clinical applications [16,17,18,19]. The mRNA vaccine requires a cold chain (−20 °C to −80 °C), which is challenging for resource-constrained settings [20]. Lyophilization as a drying technique has proven to be a compelling approach to storing and transporting mRNA vaccines under nonfreezing conditions without affecting their efficacy [21]. In a recent finding, Alejo et al. demonstrated that lyophilized mRNA-LNPs maintained stability and efficacy for a year in a refrigerated environment. They found that selecting an appropriate buffer solution and optimizing freeze-drying significantly influenced efficacy. Tris buffer has shown better transfection compared to PBS, which was assigned due to the lower leakage of RNA and its minimal influence on particle size [22]. The stability of mRNA vaccines has been mainly improved by developing carrier technologies. Carriers for mRNA delivery are usually composed of nanoparticles, which shield mRNA from degradation and ensure its stable delivery. Interest in nanocarrier-assisted mRNA delivery was sparked by the successful execution of COVID-19 mRNA vaccines (Moderna’s mRNA-1273 and Pfizer/BioNTech’s BNT162b2) [23,24,25,26]. Inspired by LNP, several other nanocarriers have been developed, aiming to prevent the degradation of mRNA and facilitate its smooth delivery [27,28,29,30,31,32]. Moreover, the success of COVID-19 mRNA vaccines has opened new possibilities for adopting this approach to combat other viral infections. It has brought a revolutionary improvement in advanced therapeutics.

This review focuses on recent developments made to deliver mRNA vaccines using various kinds of nanocarriers including LNP, polymeric nanoparticles, dendrimers nanoparticles, polysaccharide nanoparticles, peptide nanoparticles, carbon nanoparticles, metal nanoparticles, DNA nanostructures, hybrid nanoparticles, liposomes and extracellular vesicles. Attention is focused on the application of mRNA vaccines for various infections beyond COVID-19 to demonstrate the universality of mRNA vaccines, highlighting their emerging role in advanced therapeutics. The influence of carriers on enhancing the efficacy, immunogenicity, stability, and cellular uptake of mRNA vaccines has been discussed. The pros and cons of various nanocarriers are highlighted. Furthermore, we have summarized the existing challenges and proposed future research directions to achieve safe and targeted delivery of mRNA vaccines against emerging infectious diseases. This review will facilitate a deeper understanding of nanoparticle-based delivery of mRNA vaccines for emerging infections.

## 2. Lipid Nanoparticles (LNPs)

**Figure 1:**
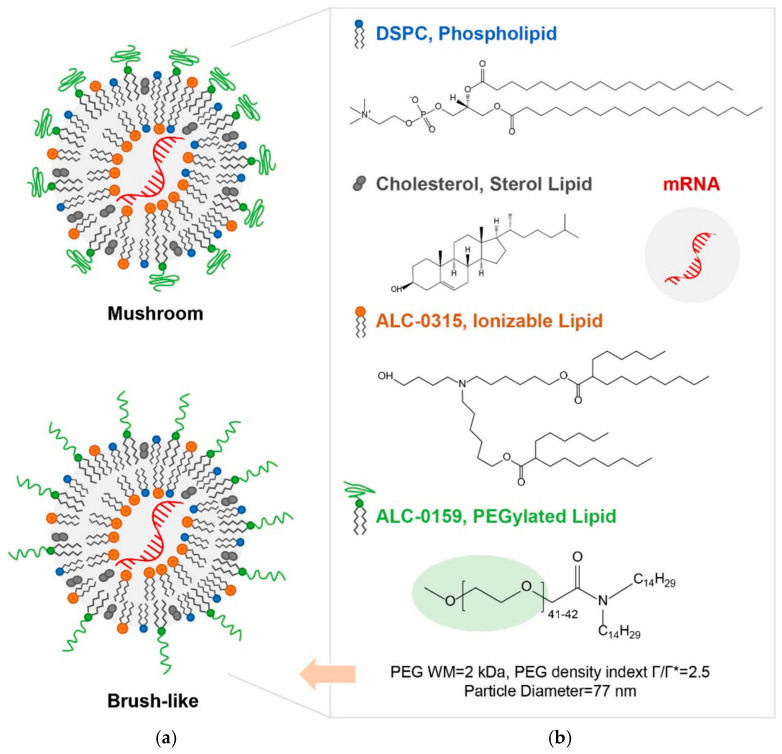
(**a**) Models of LNP and (**b**) Structures of LNP components adopted from [37].

LNPs are spherically shaped nanoscale structures. As nanocarriers, they can encapsulate mRNA, protecting it from degradation and releasing it in desired cells or tissues. They have demonstrated smooth delivery of mRNA for infections, cancer, and genetic disorders [33,34,35]. LNPs for clinical applications consist of four components: ionizable cationic lipids, cholesterol, phospholipids, and PEG-lipids [19,36]. The structures of these components are given in Figure 1. LNP promotes cellular uptake of mRNA by endocytosis.

**Figure 2:**
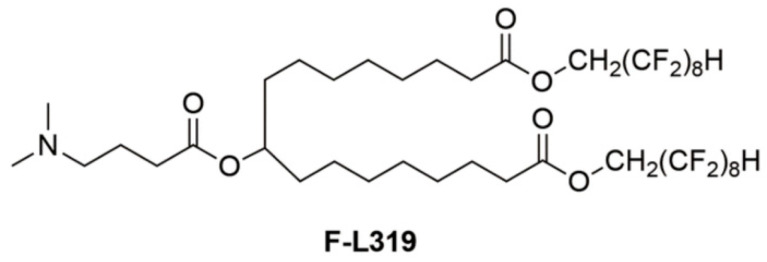
The chemical structure of fluorine modified ionizable lipid (F-L319) adopted from [38].

Ionizable LNPs in endosomes can alter their charge, facilitating the release of mRNA, which produces the target protein. This pathway promotes the delivery of mRNA to the targeted cells. Notably, the encapsulation and mRNA delivery capabilities of LNPs are greatly influenced by their size, functionalization, and charge on the surface [19]. Chemical composition can also significantly impact the delivery efficiency and efficacy of mRNA vaccines. Huo et al. have prepared a modified form of ionizable lipid by introducing fluorine in the structure referred to as “F-L319”. The chemical structure of F-L319 is given in Figure 2. Pristine structure without fluorine incorporation was referred to as “L319”. It was observed that the hybrid formulation of F-L319 and L319 with optimized proportions has significantly boosted the delivery of mRNA as compared to individual LNPs [38].

**Figure 3:**
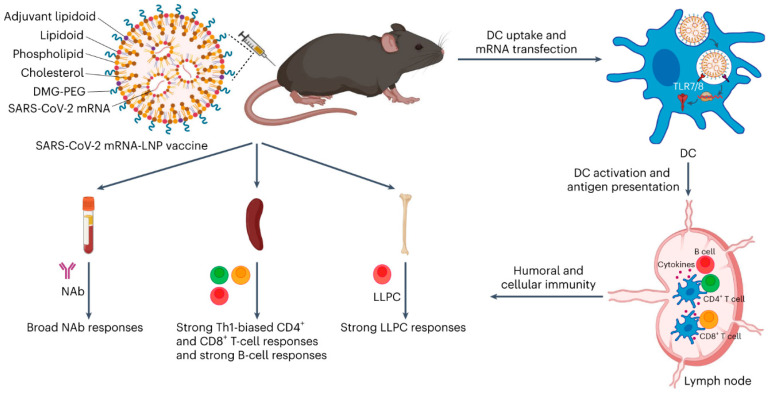
Adjuvant lipidoid-substituted SARS-CoV-2 mRNA-LNP vaccine and its mechanism of elicit immunity adopted from [39]. After injection, mRNA translated inside DCs, antigen is processed and presented, inducing adaptive immune responses.

Improvement in the delivery of mRNA by hybrid formulation has been assigned due to improvement in both mRNA loading and cellular intake. This finding highlights the important role of rationalized chemical modification of LNPs in upgrading their activity for mRNA delivery. Han et al. have revealed the integration of LNPs with adjuvant to improve the adjuvanticity of the SARS-CoV-2 mRNA-LNP vaccine [39]. It promoted mRNA delivery and enhanced Toll-like receptor 7/8-agonistic activity. This approach has generated strong T cell and B cell responses, as well as long-lived plasma cell (LLPC) responses (Figure 3).

**Figure 4:**
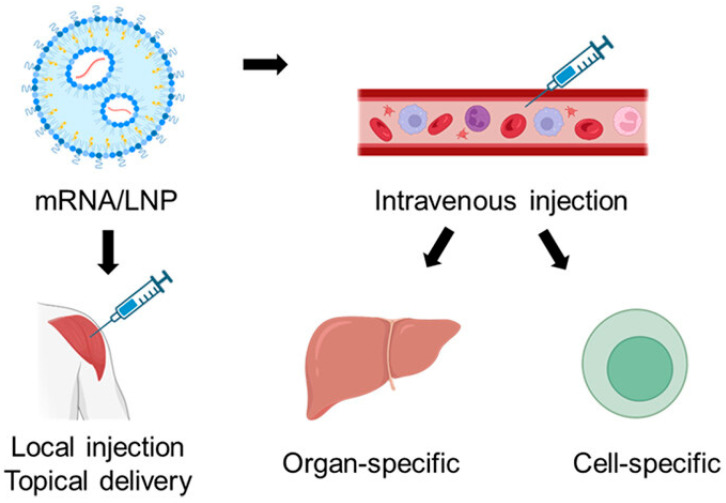
Targeted delivery of LNPs assisted mRNA vaccines adopted from [41].

This study highlights the importance of LNP modification with adjuvant lipidoid to optimize the immune response of mRNA vaccines. This concept has the potential to be extended to other vaccines to optimize immune responses. Hunter et al. highlighted machine learning techniques to achieve enhanced delivery of mRNA vaccine. Advanced cellular and endocytic profiling for intracellular delivery (ACE-ID) is integrated with LNPs for intercellular mRNA delivery [40]. In addition to protecting mRNA vaccines against degradation, LNPs provide the advantage of targeted delivery to specific cells or tissues, avoiding the side effects [41] as shown in Figure 4. Thanks to the diverse advantages of LNPs for the delivery of mRNA vaccines beyond COVID-19, these are being investigated for a diverse range of infections, including viral infections, cancer immunotherapies, metabolic disorders, and genetic disorders [42].

## 3. Polymeric Nanoparticles

Polymers are macromolecules made by repeating units of monomers. Nanoparticles made from natural and synthetic polymers are an attractive choice for delivering mRNA vaccines. The following classes of polymer nanoparticles have been most applied for mRNA vaccine delivery: esters, dendrimers, polysaccharides, and chitosan. Wilson et al. have highlighted the efficient delivery of self-amplifying mRNA (SAM) by using poly(beta-amino ester)s (PBAEs) as carriers. A significant difference was observed by comparing SAM’s intramuscular (IM) expression with and without polymer formulations. Polymer-assisted formulation has resulted in a 37-fold improvement in SAM expression. This platform has demonstrated superior immunogenicity at lower RNA doses to encode the rabies virus glycoprotein using SAM [43]. The adaptability of polymeric nanoparticles in developing mRNA clinical applications is highlighted by the capacity to tailor their chemical characteristics, which enables extended mRNA stability and biodistribution. Suberi et al. demonstrated inhalable Poly(amine-co-ester) (PACE) polyplexes nanoparticles that effectively deliver mRNA to the lungs. The PACE end was modified with PEG, and it was biodegradable with no harmful toxic effect. This platform was used to prepare mucosal vaccines against SARS-CoV-2, effectively producing cellular and humoral responses in mice [44,45]. It demonstrates the utility of PACE-mRNA-based nontoxic and inhaling complex for lung infection. Patel et al. have exploited PBAEs to design inhalable formulations for mRNA delivery to the lungs. It has consistently produced protein in the lungs without affecting other tissues. No signs of toxicity were observed. It shows the appropriateness of prepared formulations for lung infection [29].

The PBAEs are frequently applied for mRNA delivery. Their cationic characteristics promote electrostatic interaction with mRNA [46]. Meanwhile, a pH-responsive nature facilitates endosomal escape [47]. Other features making them attractive among nanocarriers include their biodegradable nature, non-toxic byproducts, lower cytotoxicity, efficient transfection, and rapid mRNA expression [48,49]. Poly (lactic-co-glycolic acid) (PLGA) is an FDA-approved biodegradable polymer with a long history of drug delivery [50]. However, they have limited interaction with negatively charged mRNA. It makes them less attractive for encapsulating mRNA. Moreover, the transfection efficiency of PLGA could be greatly improved by blending them with cationic polymers such as PEI and PBAE [46,51]. There has been an expansion in the application of polymeric nanoparticle-based mRNA vaccines for inhalable delivery to combat respiratory infections. The nasal administration route has emerged as a useful strategy for addressing threats associated with nasal and respiratory infections [52]. It is a beneficial strategy for addressing lung-related infections, such as asthma, COPD, and cystic fibrosis [53]. Ongun et al. demonstrated localized mRNA delivery to the respiratory tract through the nasal route. This work highlighted the significance of PEG-modified LNP as a nanocarrier in delivering mRNA. Optimized PEG contents were essential to balance colloidal stability and transfection efficiency [54].

**Figure 5:**
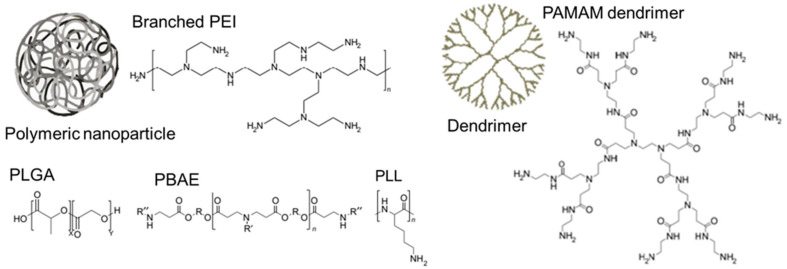
Structure of polymeric nanoparticles and dendrimer adopted from [59].

Akiva et al. have designed biodegradable polymeric nanocarriers that enable targeted delivery of mRNA to dendritic cells. It has activated antigen-specific CD8+ T cells and shown promising antitumor activity [48]. In addition to PACE polyplexes, polymeric nanoparticles such as chitosan, polylysine, and cationic polymers are investigated. These formulations can facilitate intracellular delivery by forming complexes with mRNA [55,56,57]. Figure 5 shows the structure of polymeric and dendrimer nanoparticles for mRNA delivery. Recent developments in molecularly imprinted polymers (MIP) have shown specificity in interacting with recombinant nucleocapsid protein of SARS-CoV-2 [58]. This finding highlights that polypyrrole (Ppy)-based MIP electrochemical biosensors for detecting the nucleocapsid of SARS-CoV-2 exhibit a high sensitivity of MIPs. The concept of molecular imprinting could be extended to polymeric nanoparticles for mRNA encapsulation and controlled release. It could facilitate the targeted delivery of mRNA to immune cells and enhance the efficacy of the vaccine.

## 4. Dendrimer Nanoparticles

**Figure 6:**
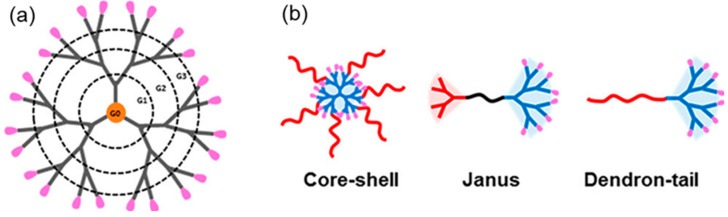
Schematic representation of dendrimer (**a**) dendrimer showing G0, G1, G2, and G3 generations and (**b**) amphiphilic dendrimers, red and blue colors representing hydrophobic and hydrophilic portions, respectively, adopted from [63].

These offer several advantages in delivering mRNA compared to other nanocarriers. Their distinctive structural characteristics make them a desirable candidate for mRNA delivery. Through efficient encapsulation, they offer enhanced protection to mRNA [60]. Highly branched, three-dimensional structures of dendrimers with tunable surface functionalities enable precise loading and delivery of mRNA. Surface groups at dendrimers facilitate the anchoring of mRNA through the formation of stable complexes. Moreover, observation of biosafety in clinical trials reflects them as a promising candidate to deliver mRNA vaccines [61,62]. Amphiphilic dendrimers are a combination of lipids and dendrimers, and they may provide the benefits of both lipids and polymers. A schematic diagram of dendrimers and amphiphilic dendrimers is shown in Figure 6 [63]. The hydrophilic portion combines RNA, whereas the hydrophobic portion can stabilize RNA. Dendrimers have multiple branching layers, indicated by their generation number. Higher-generation dendrimers have large sizes, higher branches, and more surface charges, leading to increased mRNA encapsulation and improved cellular uptake. In comparison, lower-generation dendrimers have small sizes, fewer branches, reduced surface charges, and limited interaction with mRNA, affecting encapsulation efficiency. However, higher generations may lead to cytotoxicity, necessitating the optimization of the dendrimer branching and charge density for the efficient and safe delivery of the mRNA [61,64]. The toxicity of dendrimers is due to the interaction of cationic groups with cell membranes. The surface modifications of the cationic dendrimers with neutral, anionic, or biodegradable functional groups can reduce their cytotoxicity. Modifying the dendrimers with the biodegradable core, branching units, and functional groups such as polyethers, polyether amines, and polyesters, improves biocompatibility for safety [65,66,67].

Furthermore, the dendrimers are functionalized with specific ligands for targeted delivery of mRNA. For example, dendrimers conjugated with folic acid specifically target folate receptors, which are overexpressed in cancer. Similarly, the attachment of tumor-specific antibodies with dendrimers enables targeted delivery. These cancer-targeting properties are beneficial for improving therapeutic efficacy and reducing off-target effects [68]. Despite these advantages, the complications of manufacturing dendrimers have some challenges that hinder their clinical applications. The synthesis involves multiple steps, leading to batch-to-batch variations and higher costs. Achieving high purity and maintaining mono-dispersity during mass production are essential, as their variation can affect efficacy and safety, specifically for biomedical applications [69].

Commonly adopted dendrimers for mRNA delivery include polyamidoamine (PAMAM), poly-L-lysine, and polypropylenimine [70]. Joubert et al. have demonstrated the chemical modification of PMAM- and lysine-based dendrimers to optimize the loading and delivery of mRNA. They have shown that chemical modifications improved fusogenic characteristics, buffering capacity, or mRNA condensation. Transfection activity depended on chemical composition; amino acids with a moderate basic nature of α-amino were not supportive for transfection [71]. PAMAM with branched configuration and enriched with amino groups facilitates effective anchoring of mRNA [72]. Chahal et al. have demonstrated the application of the modified dendrimers-based platform for mRNA delivery. This synthetic system produced antigens and protected against multiple infections, including Ebola, influenza, and Toxoplasma gondii [73]. Furthermore, Zhang et al. have demonstrated the application of ionizable amphiphilic Janus dendrimers (IAJDs) for the delivery of mRNA to the liver, lungs, and spleen [74]. It has been observed that the hydrophobic behavior of IAJDs has facilitated the targeted delivery of mRNA to desired organs and cells. This work shows the significance of rationalized modification-induced hydrophobicity for tailoring the mRNA delivery capabilities of dendrimers. Dendrimer-based formulations are efficiently absorbed by the numerous cells. They have the potential to be used as delivery formulations for ocular therapeutics, owing to their targeted delivery and enhanced pharmacokinetics [75].

## 5. Polysaccharide-Based Nanoparticles

Polysaccharide nanoparticles have attracted a lot of interest in delivering mRNA vaccines because of their inherent immunomodulation, biocompatibility, and biodegradable features. Naturally occurring polymers can be modified to generate nanocarriers that can encapsulate mRNA, protecting them from deterioration and promoting cellular uptake [76]. Recent research has shown the capability of polysaccharide-based nanoparticles in delivering mRNA vaccines, where the unique characteristics of these nanoparticles are vital in modulating the immune response [77]. The type of polysaccharide used in nanoparticle formulation, such as chitosan, dextran sulfate, and hyaluronic acid (HA), as well as the inclusion of adjuvant polyinosinic–polycytidylic acid (poly(I:C)), play a role in determining the immune response [78]. The mRNA encapsulation to HA nanocarriers could be challenging due to the anionic nature of HA [79]. Several strategies have been reported to overcome this issue. Djafari et al. demonstrated the HA complexation design with Poly(L-Lysine) as carriers. It has facilitated the encapsulation and delivery of microRNAs, suggesting the potential of this strategy for delivering mRNA [80]. Ryoung et al. have extended the complexation of HA with sulfur-based crosslinkers as suitable nanocarriers to enhance the stability and delivery of mRNA [81]. Myint et al. have demonstrated the fabrication of HA-based nanogels to improve the encapsulation of mRNA. Functional groups of HA can facilitate the formation of nanogels through various strategies such as crosslinking, chemical modification, or polyelectrolyte complexation. Hyaluronic acid has shown excellent colloidal stability and exhibits controlled release [82].

Electrostatic interaction between chitosan and mRNA can prevent the degradation of mRNA. Moreover, its stimuli-responsive nature facilitates the controlled release of encapsulated mRNA [83]. Chitosan, being a biocompatible polymer, offers the advantage of reducing carrier-associated toxicity. For instance, Garcia et al. have demonstrated that adding chitosan-N-arginine (CSA) into DOTAP lipid and DOPE helper lipid has reduced the cytotoxic effects. Besides that, it also improved transfection in HeLa and HEK293T cell lines [84]. Chitosan-based formulations have the potential to be administered through mucosal delivery, which is a needle-free route [85,86,87]. However, certain limitations exist in exploiting chitosan as a nanocarrier to deliver mRNA. These include limited solubility of chitosan in physiological pH, difficulty tailoring uniformity in chitosan-based nanoparticles, and poor mechanical strength, which may cause burst release of mRNA [88]. Other challenges associated with chitosan nanocarriers include scalability, stability, and nanotoxicity. It can be challenging to maintain batch-to-batch uniformity of chitosan nanocarriers [55]. Chemical modifications of chitosan and making a hybrid with crosslinkers can improve the stability and delivery of mRNA [89]. Nonetheless, more investigations are needed to unfold the mechanisms involving polysaccharide-based nanoparticles in mRNA vaccine delivery.

## 6. Peptide-Derived Nanoparticles

**Figure 7:**
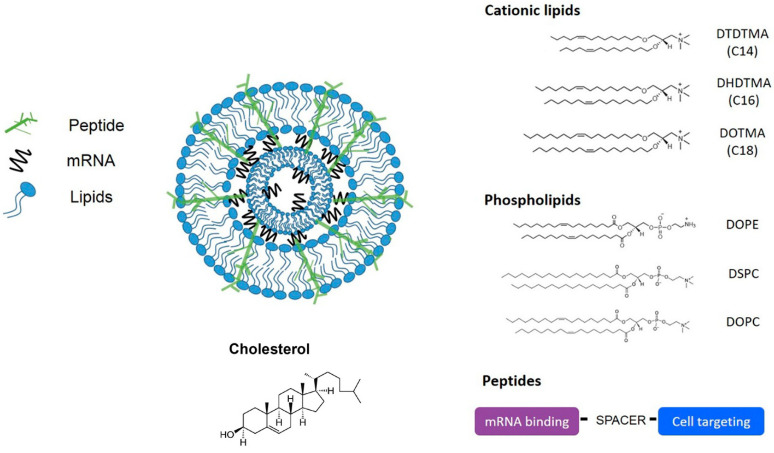
Structure of lipid peptide nanocomplex for mRNA vaccine delivery [97].

Self-assembled amphipathic peptides can effectively complex and protect mRNA, facilitating cellular uptake and intracellular delivery [90]. One advantage of these nanoparticles is their ability to be customized for specific targeting and enhanced cellular internalization. Peptide-driven mRNA delivery offers unique advantages owing to their diversity [8]. By adjusting the composition of amino acids, diverse functionalities can be incorporated for improved mRNA delivery. These functionalities include enhanced endosomal escape efficiencies, targeted delivery to dendritic cells (DCs), proficient antigen presentation, and targeted lung delivery. For instance, cell-penetrating peptides extended protein expression through stabilizing mRNA intracellularly [91]. The novel cell-penetrating peptides NF424 and NF436 have demonstrated exceptional efficacy in specifically targeting mRNA delivery to spleen tissue [92]. Certain peptide sequences have demonstrated efficacy in promoting binding and uptake by antigen-presenting cells. The arginine-rich peptides have been utilized to formulate mRNA nanocomplexes capable of inducing cytotoxic T cell immune response [93]. Preclinical studies have validated peptide-driven LNPs’ efficacy in delivering mRNA vaccines to the neural retina [94]. Additionally, peptide-based nanocarriers have been investigated to deliver mRNA for tumors and stimulate antigen-specific immune responses against cancer [95]. These findings hold profound significance for the progression of cancer immunotherapy, presenting a promising avenue for activating cytotoxic T cells via mRNA encoding tumor antigens [92]. Figure 7 shows the structure of the lipid–peptide complex. As a biopolymer, albumin has a higher degree of biocompatibility, making it an attractive choice for delivering mRNA. Moreover, the anionic nature of albumin facilitates solubility in water [96]. Several studies have investigated the potential of albumin-based nanoparticles in therapeutics. Cai et al. have exploited albumin as a protein-based nanocarrier for delivering mRNA.

They have modified bovine serum albumin with poly-L-lysine and integrated it with macrophage cell membranes. It has prevented the mRNA from degrading and improved its transfection efficiency [98]. Kida et al. have reported human serum albumin-based nanobubbles as an effective tool to deliver mRNA, bypassing the need for stabilizers. The nanobubble structure was retained during lyophilization [99]. In another study, albumin has been combined with glutaraldehyde as a cross-linker for improving stabilization. However, careful consideration is required to avoid the associated risk of increasing toxicity [100,101]. Endosomal escape is a key limiting factor for peptide-based mRNA delivery systems. Several strategies, such as chemical modification of peptides and the designing of pH-responsive peptides, have been reported to overcome this obstacle.

Zhang et al. have designed poly(glutamic acid) nanoparticles as pH-responsive carriers that can enhance the antigen lysosomal escape. Structural change of *α*-PGA NPs in acidic conditions promotes membrane fusion and improves cellular immunity [102]. Xu et al. have further investigated PEGylation of peptide nanoparticles for pH-responsive release of mRNA for pulmonary delivery. They observed that the PEG chain of 12 monomers substantially enhanced mRNA delivery compared to the pristine peptide. In contrast, a longer PEG chain has reduced transfection efficiency. It highlights the significance of tailoring chemical modification of peptide nanoparticles for enhancing pH-dependent controlled release of mRNA and its cellular uptake [103]. In a recent finding, Zhang et al. highlighted the significance of introducing peptides to auxiliary lipids for enhancing endosomal escape. They revealed that the synergetic effect of the KHL/DOTAP complex has significantly enhanced endolysosomal escape (about 10-fold) compared to individual components [104].

## 7. Carbon-Based Nanoparticles

**Figure 8:**
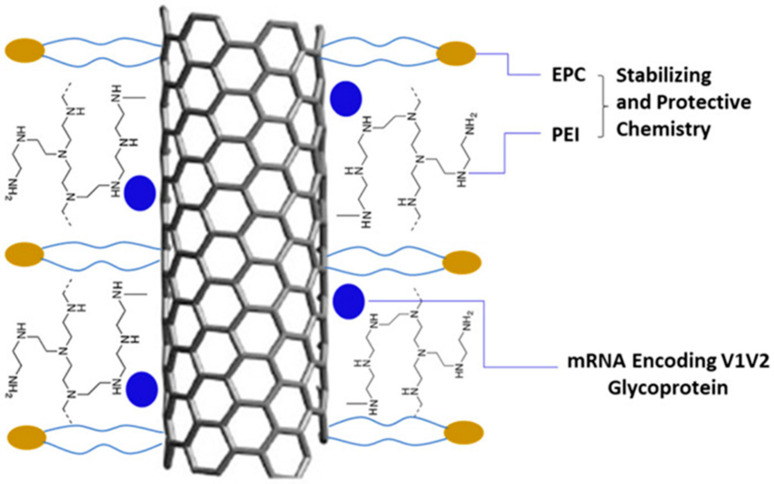
Surface chemistry of CNT for mRNA delivery adopted from [105].

Carbon-based nanoparticles have great potential to deliver mRNA vaccines owing to their distinctive characteristics. Carbon nanotubes (CNT) have been studied as a platform (NanoVac) for delivering mRNA encoding the HIV-1 glycoprotein V1V2 region. Surface-modified NanoVac facilitated mRNA loading, cellular entry, and endosomal escape, resulting in efficient transfection. Immunogenicity studies using rabbit and humanized mouse models demonstrated that NanoVac induced robust immune responses for HIV-1 antigen and favorable safety and stability profiles [105]. Furthermore, NanoVac effectively preserved mRNA from degradation and reduced efforts to maintain a cold chain during vaccine deployment [105]. A schematic diagram of CNT for mRNA delivery is given in Figure 8. Recently, by screening different amphiphilic carbon dots, researchers found that O12-Tta-CDs have promoted the delivery of mRNA.

The amphiphilic nature of O12-Tta-CDs, with the hydrophobic alkyl chains and the ionizable carboxylic groups from the thiophene derivative, enabled efficient binding and delivery of mRNA to immune cells. It has actively prevented tumor development by inducing T cell infiltration [106].

## 8. Metal-Based Nanoparticles (MNPs)

**Figure 9:**
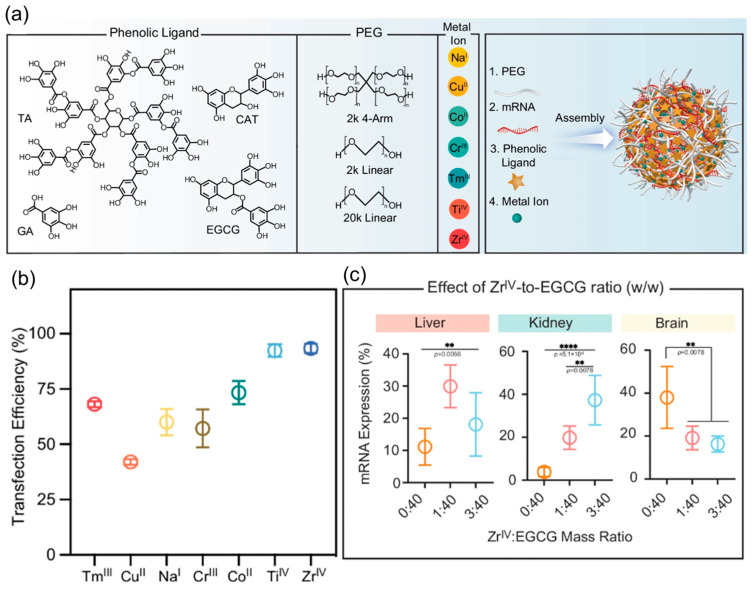
(**a**) Schematic diagram for the synthesis of mRNA-MPN NPs through metal–phenolic-mediated assembly of PEG, mRNA, phenolic ligands, and metal ions, (**b**) transfection efficiency of mRNA-MPN NPs assembled with various metal ions, and (**c**) mRNA expression in harvested organs using MPN NPs with different Zr^IV^-to-EGCG mass ratios adopted from [108]. Analysis was carried out using one-way ANOVA or one-way ANOVA with Tukey’s multiple comparisons test. In liver (** *p*  =  0.0066), kidney (**** *p*  =  5.1 × 10^−5^, ** *p*  =  0.0078), and brain (** *p*  =  0.0078).

Recent research highlights the advantages of MNPs in mRNA delivery. For example, dendrimer-coated gold nanoparticles combined with folic acid modification have resulted in better mRNA stability and transgene expression, offering effective protection of mRNA against nucleases and elevated levels of gene expression [107]. As mRNA therapeutics advance, integrating MNP-based delivery systems presents significant potential to address mRNA instability and inefficient cellular uptake challenges, ultimately promoting mRNA vaccine delivery. Gu et al. have investigated the suitability of metal–phenolic networks (MPN) nanoparticles to deliver mRNA for expressing protein in different organs. They have combined poly(ethylene glycol)-polyphenol with a range of metals, as shown in Figure 9. Among these, ZrIV and TiIV have demonstrated higher transfection efficiency. Linear PEG, selected mRNA, epigallocatechin gallate (EGCG), and ZrIV at a mass ratio of 100:1:100:2.5 have shown effective protein expression for the liver, kidney, and brain. The above finding highlights the potential of metal and metal–organic-based nanoparticles for mRNA delivery [108]. Although black phosphorus (BP) exhibits a non-metallic character, BP nanosheets modified with polyethyleneimine have been investigated as a carrier to deliver mRNA. Yang et al. have observed the contribution of BP to facilitate immune activation and antibody production. It highlights the potential of phosphorous-based nanocomposites as vehicles for delivering mRNA [109].

## 9. DNA Nanostructures

**Figure 10:**
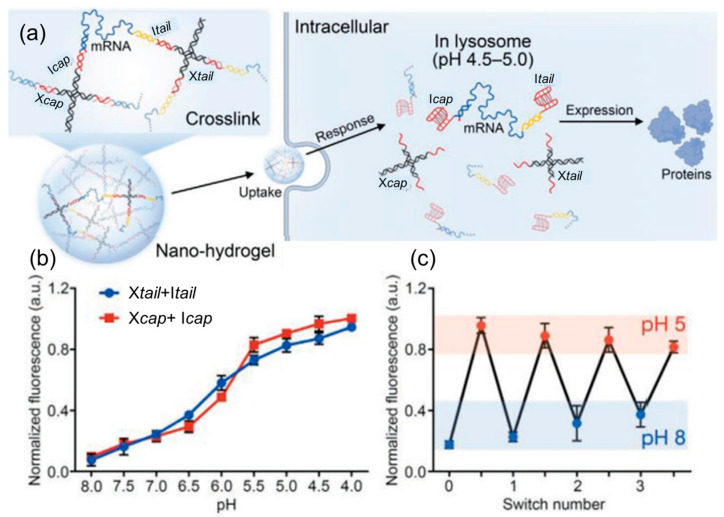
(**a**) Schematic representation of DNA nanohydrogel-assisted mRNA delivery and its intracellular pH-responsive release, (**b**) fluorescence intensity analysis of X_tail_ + I_tail_ and X_cap_ + I_cap_ under different pH conditions, and (**c**) switch cycles of the X_tail_ + I_tail_ and X_cap_ + I_cap_ between a of pH 5.0 and 8.0, adopted from [113].

These are crafted from DNA molecules to encapsulate and safeguard mRNA payloads, facilitating their precise delivery to cells. The major benefits of DNA-based carriers are providing precise control of mRNA loading, cellular uptake, and endosomal escape. Furthermore, I-motif and DNA triplet structures are useful for sensing pH, facilitating the controlled release of RNA, specifically in a cellular environment with acidic conditions [110]. Recently, a variety of DNA nanostructures, including DNA tetrahedral nanostructures [111], DNA nano suitcases [112], DNA nano hydrogels [113], and DNA origami [114], have been designed for mRNA delivery. DNA-based hydrogel has effectively released mRNA from cells, which is on par with liposomes but with better biocompatibility [113]. The authors have demonstrated that pH has played a crucial role in delivering mRNA by nano hydrogel. A schematic diagram of nano hydrogel fabrication is presented in Figure 10a. In nano hydrogel X-shaped DNA scaffolds facilitate crosslinking of DNA with mRNA. Under acidic pH conditions (4 to 4.5) in the lysosome, it dissociates and releases mRNA to express the protein. The pH-dependent response of nano hydrogel containing Cy5 is given in Figure 10b, which shows high fluorescence intensity under pH 4.0 to 4.5. It shows the disintegration of nano hydrogel and the release of mRNA. Figure 10c shows quenching and recovery of fluorescence under alkaline and acidic pH. Another study by Li et al. reported the creation of a DNA-based nanomachine that responds to thermal and enzymatic stimuli. The main component of this nanomachine was polythymidine acid (Poly-T) functionalized poly(N-isopropylacrylamide) (DNA-PNIPAM). The core of DNA-PNIPAM was coated with a shell of 2-hydroxypropyltrimethyl ammonium chloride chitosan (HACC) [115]. The HACC shell facilitated cellular uptake and protected mRNA from enzymatic degradation.

**Figure 11:**
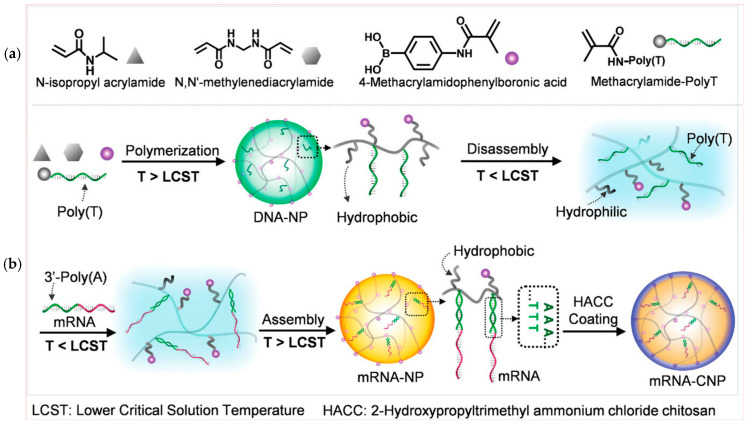
Molecular design and preparation of the nanomachine for mRNA delivery. (**a**) The monomers and their corresponding legends used in the polymerization for the preparation of DNA-integrated nanoparticles (DNA-NPs) and (**b**) the synthesis and thermal-responsive phase transition of PNIPAM-based nanostructure adopted from [115].

It has a lower critical solution temperature (LCST) of about 32 °C in an aqueous environment. It switched to hydrophilic and hydrophobic states below and higher than the LCST, which facilitated the nanomachine to encapsulate mRNA. The molecular design of the nanomachine and the LCST-dependent responsiveness are shown in Figure 11. The charge on the nanomachine was regulated by adjusting the concentration of HACC; without HACC, it showed a negative charge, whereas with 1 and 2 mg/L of HACC, cationic behavior was observed with a zeta potential of +11.72 mV and +25.83 mV, respectively. The average size of the nanomachine observed through TEM analysis was about 154 nm, which is appropriate for cellular uptake. The introduction of a DNA-based nanosystem has addressed the problem of cells that are hard to transfect and have high levels of glutathione (GSH) [116]. To improve mRNA transfection, this approach uses siRNA that promotes ribosome biogenesis. It loads siRNA and mRNA effectively by utilizing sequence-specific molecular recognition and heat responsiveness. Furthermore, intracellular GSH-responsive disassembly promotes siRNA-mediated GSH depletion and optimized mRNA and siRNA release.

The latest finding by Hu et al. has unfolded DNA origami for effective delivery of Smad4 mRNA. Only two circular RNA strands are used as “staples” in this flexible RNA origami structure to bind the mRNA, leaving a large portion in a single-stranded, active form. This adaptable structure permits efficient delivery of mRNA while retaining the ability of ribosomes to recognize and translate it into the cytoplasm. In comparison to other carriers, the researchers showed that this mRNA nanolantern had comparable mRNA delivery efficacy, reduced toxicity, and superior RNase resistance [117]. Rationalized tuning of functional features of DNA nanostructure may improve the stability and delivery of mRNA. The development of these origami-based delivery methods may increase efficacy.

## 10. Hybrid Nanoparticles

**Figure 12:**
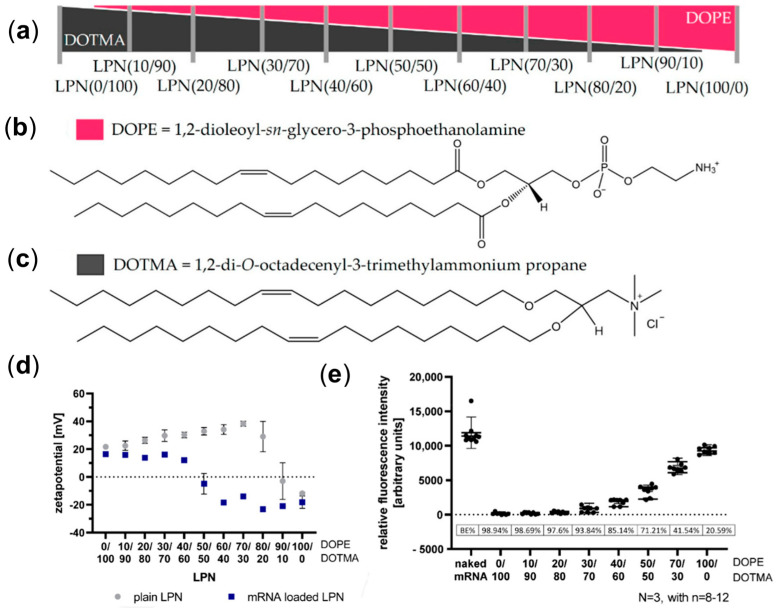
(**a**) Composition tuning of DOPE/DOTMA, (**b**,**c**) chemical structures of DOPE and DOTMA, (**d**) composition dependent zeta potential, and (**e**) corresponding relative fluorescence intensity adopted from [120].

Hybrid nanoparticles provide notable advantages for mRNA delivery by combining the benefits of different materials. Adopting inorganic-organic hybrid nanostructures by incorporating inorganic components like silica or gold nanoparticles into organic matrices like lipids or polymers provides a versatile opportunity [118]. For instance, mesoporous silica-lipid hybrid nanoparticles have been devised for mRNA delivery, showcasing enhanced encapsulation [119]. Lipid–polymer nanoparticles (LPN) as hybrid carriers for mRNA delivery are given in Figure 12 [120]. Zeta potential of LPN with and without loading mRNA with respect to the relative proportion of phospholipid 1,2-dioleoyl-*sn*-glycero-3-phosphoethanolamine (DOPE) and cationic lipid 1,2-di-*O*-octadecenyl-3-trimethylammonium propane (DOTMA) are shown in Figure 12a–c. The zeta potential of LPN was observed in the positive range and increased by increasing the DOPE proportion, reaching a maximum at DOPE 70%. It indicated suitability for encapsulating negatively charged mRNA. Increasing DOPE higher than 90% has shown the appearance of a negative charge on LPN, which may not be appropriate for mRNA loading because of lacking electrostatic interactions. This is evident from the trend given in Figure 12d. The LPN with 40% DOPE showed a binding efficiency of around 85.14%, and it decreased drastically to 20.59% as DOPE increased to 100% (Figure 12e).

Hybrid nanoparticles provide mRNA protection and efficient encapsulation via the lipid while imparting stability, controlled release, and potential surface functionalization through the polymer. Li et al. introduced an N1, N3, N5-tris(2-aminoethyl)benzene-1,3,5-tricarboxamide (TT) derived lipid-like nanomaterial (TT3-LLN), which has shown effective mRNA delivery capabilities [121]. Biodegradable and biocompatible polymers are integrated with TT3-LLNs to upgrade delivery. Zhao et al. have introduced hydrophilic polymers, including dextran and HA, and hydrophobic polymers, including poly lactic-*co*-glycolic acid (PLG) and poly(d,l-lactic acid) (PLA) with TT3 LLN lipid-like nanoparticles [122]. The zeta potential of polymers modified with TT3 LLN was in the positive range except for HA. Furthermore, this finding concluded that hydrophobic polymer modification, especially PLG, has shown better transfection than hydrophilic polymers. Among various formulations, PLGA4-7 LPNs were observed as an optimized formulation for significantly improving the delivery efficiency of mRNA. The size, zeta potential, and encapsulation efficiency of eGFP mRNA-loaded PLGA4-7 LPNs were 287.2 nm, 22.7 mV, and 98.87%, respectively. It demonstrates the potential of hydrophobic modification of TT3-LLNs for efficient delivery of mRNA in human cell lines in advanced biomedical applications.

Recently, Yadava et al. introduced hybrid lipid nanocapsules (hLNCs) composed of kolliphore HS15, labrafac lipophile WL1349, DOPE, a conjugate of oleic acid (OA), and polyethylenimines (PEI) with three different molecular weights (PEI 0.8, 1.8, and 25 kDa). These hLNCs were stable under different pH ranges. In vitro and in vivo analyses have reflected the effective transfection efficiency of mRNA-loaded hLNCs [123]. Zeta potential can significantly affect cellular uptake and transfection capability. The zeta potential of OA-PEI hLNCs was greatly influenced by the molecular weight. A gradual increase in the molecular weight of PEI has resulted in an increased positive charge due to a higher proportion of amine groups. The transfection efficiency trend reflected that lower molecular weight has shown better performance due to their optimized ability to release mRNA. Other hybrid nanoparticles composed of DOTAP–polymer have shown promise for mRNA delivery to treat cancer, infections, and genetic disorders [35].

## 11. Liposomes and Extracellular Vesicles

In order to improve mRNA stability and immunogenicity for diverse therapeutics, current investigations and endeavors seek to effectively optimize the liposomes and extracellular vesicles (EV) [124]. To overcome the difficulty of inefficient delivery of mRNA, liposomes have become popular as delivery vehicles. Duskunovic et al. have examined the integration of dextran-based nanogel particles, which were modified with oligoarginine peptide (DNPR9), and incorporated into liposomes (LipoDNPR9). These particles improve the effectiveness of mRNA delivery, demonstrating notable enhancements in cellular uptake and transfection rates [125].

**Figure 13:**
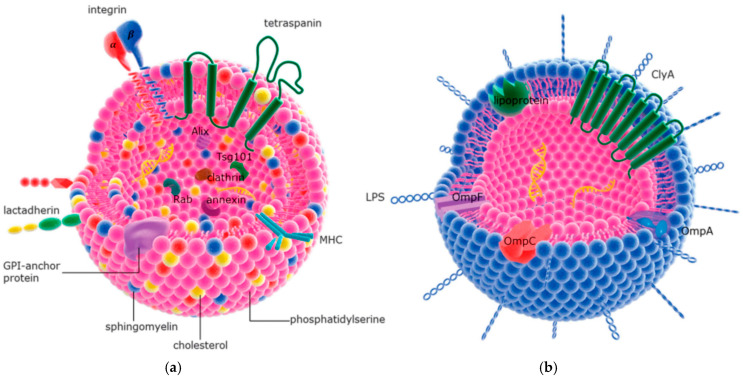
Molecular structure and composition of extracellular vesicles derived from mammalian cells (**a**) and gram-negative bacteria (**b**) adopted from [131].

The zeta potential of LipoDNPR9 was observed as +34 mV, greater than the +26 mV observed for liposome. Authors have assigned this increase in zeta potential as a potential reason for cellular uptake. Vysochinskaya et al. have investigated the influence of liposome components and lipid-to-mRNA proportion on mRNA transfection by designing cationic liposomes [126]. They found that lipoplexes with an amino-to-phosphate group ratio of 10/1 showed the highest loading and transfection efficiency. The zeta potential of mRNA-eGFP and mRNA-FLuc was observed as >+30 mV, which is crucial for cellular uptake. Pollard et al. reported mRNA encoding HIV-1 antigen Gag, complexed with the cationic lipid DOTAP/DOPE. It induced antigen-specific humoral and cellular immunity [127]. Tane et al. have reported the significance of the modified ethanol injection approach in enhancing mRNA delivery with mRNA lipoplexes. They have evaluated 18 different types of mRNA lipoplex formulations [128]. Authors observed that mRNA lipoplexes LP-DC-1-16/DOPE and LP-DC-1-12/DOPE have shown high protein expression. The zeta potential and size of LP-DC-1-16/DOPE were 28.3 mV and 243.4 nm. Whereas th ezeta potential and size of LP-DC-1-12/DOPE were 35.9 mV and 170 nm. Extracellular vesicles (EVs), like exosomes, showcase promising nanocarriers that can be used to deliver mRNA. They are attractive substitutes for synthetic nanoparticle formulations because of their natural capacity to fuse with target cells and deliver mRNA. The molecular structures of EV are highlighted in Figure 13. Yang et al. have demonstrated the effectiveness of mRNA loading and regulating its release from EVs. The low-density lipoprotein receptor (*Ldlr*) mRNA was encapsulated into Vs by using MS2 bacteriophage coat protein (CD9-MCP) fusion protein. The average diameter of EV^CD9-MCP^ was observed as 134.1 ± 2.6. Authors have reported enhanced loading and controlled release of mRNA from EVs [129]. Pomatto et al. have applied plant-based Citrus sinensis EVs as carriers to deliver mRNA vaccines for SARS-CoV-2 [130]. The mRNA-loaded EVs have an approximate size of <100 nm, which was retained after lyophilization. Moreover, loaded mRNA has demonstrated elongated stability at ambient conditions for at least 1 year after lyophilization.

Furthermore, Li et al. have highlighted that RNA-binding proteins like L7Ae have been rapidly adsorbed and delivered mRNA antigens when integrated with bacteria-derived outer membrane vesicles (OMVs), resulting in an effective response against tumors [132]. Such developments highlight the potential of EVs as powerful carriers to deliver mRNA and improve its stability.

## 12. Challenges and Opportunities

Obstacles in existing mRNA delivery include the requirement of the cold chain, targeted delivery of multiple doses, and possible side effects [133]. Confronting these obstacles may enable mRNA vaccines to be more effective and accessible [134]. Selecting a suitable delivery method is essential for the effective administration of mRNA vaccines. The mRNA molecules must cross the lipid bilayer to access the cytoplasm for subsequent translation into functional proteins. Various carriers have been created for mRNA delivery, such as protamine, nanoemulsions, dendrimers, cationic polymers, polysaccharides, and LNPs [135]. Surface modifications of the nanoparticles with ligands can further facilitate the targeted delivery of mRNA [136,137,138]. It may lower the required dosage of mRNA while reducing the side effects to non-targeted cells [139]. However, efforts should be made to optimize mRNA vaccine development by exploring controlled-release and non-invasive administration strategies to improve safety and efficiency [140]. Optimization of nanocarriers for delivery could play a crucial role [141]. Formulation parameters (composition, particulate size, charge, and ligand modification) can significantly impact the delivery of mRNA vaccines. Despite various efforts, the delivery efficiency of LNPs remains suboptimal. A major challenge limiting transfection efficiency is the entrapment of nanoparticles in endosomes following endocytosis. Modifying the surface with a ligand could be beneficial for cellular uptake. Hybrid formulations leverage the benefits of both lipid and polymer structures at once [129,142]. It is evident that the delivery route significantly influences the dissemination of vaccines in organs, expression rate, and vaccine effectiveness.

Recently, noninvasive routes, including oral, nasal, pulmonary, ocular, and transdermal for mRNA delivery, have been explored. These routes provide benefits of self-administration, lower health care costs, minimized risk of blood-borne infections, and have the potential for mass vaccination [143]. While exploring the benefits of oral administration, Abramson et al. have developed robotic pills composed of branched hybrid poly(β-amino ester) mRNA nanoparticles. Oral intake of pills bypassed natural barriers in the gastrointestinal tract and injected mRNA nanoparticles directly into the stomach submucosa [144]. Benetti et al. have highlighted nasal delivery of mRNA. They have adopted chitosan as a carrier to deliver mRNA due to its biocompatible nature and mucoadhesive properties. Preclinical studies showed promising local immune responses without generating systemic antibody responses, revealing its potential for nasopharyngeal immunization [145]. Intramuscular administration is a widely applied approach for COVID-19 vaccines, as it effectively stimulates immune responses. However, an alternate injection route, for instance, intranasal vaccine administration, has the potential to stimulate mucosal immunity. This route is gaining attention as an emerging strategy to deliver mRNA vaccines [146]. Furthermore, the transdermal route is a promising strategy for delivering the vaccines through microneedles. Puigmal et al. have developed Poly(β-amino ester)-based carriers for vaccine delivery. Using this strategy, cell-specific transfection has been observed by integrating Poly(β-amino ester)s with oligopeptide chains. It offers a unique opportunity to exploit peptide-based systems for transdermal delivery of mRNA [147]. Ocular delivery of mRNA is a promising route among non-invasive delivery approaches. Sauvage et al. have exploited the use of polydopamine nanoparticles for delivering mRNA. In this work, irradiation has played an important role in heat generation, facilitating the formation of nanobubbles and intercellular delivery [148].

The nanoparticle-assisted delivery of the mRNA vaccine has made significant improvements in terms of stability and controlled release. However, concerns regarding immunogenicity and over-activation of innate immune responses may cause undesired side effects. Hence, it is important to carefully evaluate the immunogenic impact of nanocarrier-assisted mRNA delivery [149]. Because of compositional complexity, it could be hard to distinguish that the onset of innate immune response is either due to mRNA or LNP [150]. Alameh et al. have highlighted that ionizable lipid nanoparticles are responsible for proinflammatory cytokines [151]. Dimethyldioctadecylammonium bromide (DDAB)-based quaternary ammonium lipids can accelerate innate immune responses. It acts as an immunological adjuvant [35]. Cationic lipid carriers may cause activation of TLR2 and TLR4 proteins and produce proinflammatory cytokines [152]. Hence, there are concerns about applying them as nanocarriers for delivering mRNA. Besides immunogenicity issues linked with lipid nanocarriers, they can also accelerate autoimmune reactions. It is notable that size, charge, administration route, and choice of adjuvants can greatly influence autoimmune reactions [153]. Although the clear mechanism is unknown, clinical findings have indicated autoimmune hepatitis post-COVID-19 vaccination. It has been associated with dominant T cell immunity [154,155,156,157]. Moreover, Chavda et al. have discussed adjuvant-induced autoimmunity and the development of autoimmune diseases by vaccination [157].

The challenge of ultra-cold temperature storage for mRNA vaccines discourages its mass distribution. Current RNA vaccines require a temperature of −20 °C or −80 °C, which hinders global distribution. To address this issue, research is focusing on improving the stability and adaptability of delivery systems. Recently, lyophilized nanostructured lipid carriers (NLCs) have enhanced stability under less stringent storage conditions. It could revolutionize vaccine distribution by enabling stockpiling and rapid deployment in resource-constrained areas [158]. In another finding, Li et al. have applied a mixture of lyoprotectant containing sucrose, trehalose, and mannitol to lyophilize mRNA-LNPs. It has shown stability at 2–8 °C without compromising the immunogenicity [159]. Lamoot et al. have investigated lyophilization of mRNA LNP by introducing 20% *w*/*v* of sucrose as cryoprotectants. They observed that mRNA-LNP re-dispersed after lyophilization and retained structural stability. This approach has shown a negligible influence on particle size and zeta potential. In contrast, colloidal stability was damaged without sucrose, affecting transfection efficiency. This study highlights the significance of cryoprotectants during lyophilization [160]. Ai et al. have optimized a lyophilization process with less residual water to achieve the long-term stability of mRNA-LNPs. They have demonstrated that LyomRNA-Omicron enhanced thermostability at 25 °C for up to 6 months [161]. In another study, Wan et al. have shown mannose modification of PEG lipids for circular RNA vaccine delivery. It has sustained stability after lyophilization without negatively affecting immunogenicity and specificity [162]. This finding highlights the significance of mannose modification of nanocarriers in preserving LNPs during the freeze-drying process. Furthermore, innovative approaches such as kinetic modeling have been proposed to evaluate the thermal stability of mRNA vaccines and their deployment to low-resource settings [163].

Clinical translation of an innovative delivery platform faces certain challenges. These include safety and efficacy concerns, complexity in clinical trials, production scalability, and complicated regulatory requirements [164,165]. A fundamental concern in this regard is the risk of the unintended immune response or toxicity induced by nanoparticles. It necessitates extensive investigations to confirm their safety and biocompatibility. Careful design of nanoparticles as delivery tools is essential to avoid mRNA degradation and prevent accumulation in off-targeted sites [154,166]. Clinical trials of nanoparticle-based mRNA delivery face complexity as they need a dual assessment of both mRNA and nanoparticles. Before clinical investigations, comprehensive preclinical evaluations should be thoroughly performed to ensure the safety and efficacy of such formulations [167]. However, due to insufficient information on immunogenicity and toxicity, regulatory approval of nanoparticles for delivery of mRNA could be complicated and time-consuming [168]. Although immense efforts have been made, we are still in the infancy of understanding potential mRNA vaccine delivery systems. In the future, the outcome of the clinical trials would shed light on deeper insight into nanocarrier systems. It can facilitate better administration of various delivery methods of the mRNA vaccine platform [169]. Efforts should focus on increasing the production capacity of carriers and adjuvants. Selecting appropriate routes of administration and elucidating the immune pathways. Improving mRNA delivery systems is key to expanding the potential of mRNA therapeutics.

## 13. Conclusions

In conclusion, advancements in mRNA vaccine delivery have significantly improved the therapeutic effectiveness. Nanocarriers have made a vital contribution to the safer delivery of mRNA vaccines to targeted cells. It has improved vaccine efficacy while minimizing the off-target side effects. Surface modifications of nanoparticles have been found useful in enhancing cellular uptake of mRNA vaccines. Hyper-branching of dendrimers through large surface areas can promote the loading capability of mRNA. Nanocarriers based on biopolymers such as chitosan, albumin, and DNA are biocompatible in nature and minimize the risk of toxicity. Despite these advancements, several challenges remain to be solved, including optimizing immunogenicity, reducing toxicity, and addressing cold storage requirements. Some nanocarriers may have safety concerns; these may provoke unintended immune responses that lead to inflammation. Ongoing research and development are crucial for overcoming these obstacles and enhancing delivery strategies. Rationalized modification of nanoparticles is indispensable for mRNA delivery to specific cells or tissues. Engineering of stimulus-responsive nanoparticles may ensure the controlled release of mRNA. Furthermore, advancement in biocompatible nanoparticles could be useful to overcome the challenges of undesired immune responses. Enhancing the mRNA payload of nanocarriers could minimize the injectable quantity of nanoparticles. It can significantly reduce the risk of toxicity and side effects associated with nanoparticles. Rigorous preclinical evaluations and clinical trials are needed to ensure the safety of nanoparticle-based mRNA formulations. As the field progresses, improvement in delivery systems holds immense potential to transform mRNA therapeutics.
